# Cortical tracking of formant modulations derived from silently presented lip movements and its decline with age

**DOI:** 10.1093/cercor/bhab518

**Published:** 2022-01-22

**Authors:** Nina Suess, Anne Hauswald, Patrick Reisinger, Sebastian Rösch, Anne Keitel, Nathan Weisz

**Affiliations:** Department of Psychology, Centre for Cognitive Neuroscience, University of Salzburg, Salzburg 5020, Austria; Department of Psychology, Centre for Cognitive Neuroscience, University of Salzburg, Salzburg 5020, Austria; Department of Psychology, Centre for Cognitive Neuroscience, University of Salzburg, Salzburg 5020, Austria; Department of Otorhinolaryngology, Head and Neck Surgery, Paracelsus Medical University Salzburg, University Hospital Salzburg, Salzburg 5020, Austria; School of Social Sciences, University of Dundee, Dundee DD1 4HN, UK; Department of Psychology, Centre for Cognitive Neuroscience, University of Salzburg, Salzburg 5020, Austria; Department of Psychology, Neuroscience Institute, Christian Doppler University Hospital, Paracelsus Medical University, Salzburg 5020, Austria

**Keywords:** low-frequency speech tracking, MEG, multisensory processing, visual speech processing

## Abstract

The integration of visual and auditory cues is crucial for successful processing of speech, especially under adverse conditions. Recent reports have shown that when participants watch muted videos of speakers, the phonological information about the acoustic speech envelope, which is associated with but independent from the speakers’ lip movements, is tracked by the visual cortex. However, the speech signal also carries richer acoustic details, for example, about the fundamental frequency and the resonant frequencies, whose visuophonological transformation could aid speech processing. Here, we investigated the neural basis of the visuo-phonological transformation processes of these more fine-grained acoustic details and assessed how they change as a function of age. We recorded whole-head magnetoencephalographic (MEG) data while the participants watched silent normal (i.e., natural) and reversed videos of a speaker and paid attention to their lip movements. We found that the visual cortex is able to track the unheard natural modulations of resonant frequencies (or formants) and the pitch (or fundamental frequency) linked to lip movements. Importantly, only the processing of natural unheard formants decreases significantly with age in the visual and also in the cingulate cortex. This is not the case for the processing of the unheard speech envelope, the fundamental frequency, or the purely visual information carried by lip movements. These results show that unheard spectral fine details (along with the unheard acoustic envelope) are transformed from a mere visual to a phonological representation. Aging affects especially the ability to derive spectral dynamics at formant frequencies. As listening in noisy environments should capitalize on the ability to track spectral fine details, our results provide a novel focus on compensatory processes in such challenging situations.

## Introduction

Understanding speech is a multisensory process that requires diverse modalities to work together for an optimal experience. Congruent audiovisual input is especially crucial for understanding speech in noise (Sumby and Pollack 1954; Crosse et al. 2016), highlighting the importance of visual cues in speech processing studies. One hypothesis is that activation from visual speech directly modulates activation in auditory cortex although the results have been mixed and many questions remain unanswered (Bernstein and Liebenthal 2014; Keitel et al. 2020). One important question is with respect to the nature of the representation in the visual cortex and whether it is strictly visual or already tracks acoustic information that is associated with the visual input (for nonspeech stimuli, see, e.g., Escoffier et al. 2015). A first approach to address this hinted that occipital activation elicited by silent lip-reading also reflects the dynamics of the acoustic envelope (O’Sullivan et al. 2017). Conclusive evidence that the visual cortex is able to track certain aspects of speech by visual cues alone comes from a recent study by Hauswald et al. (2018). Evidently, it has been shown that visual speech contributes substantially to audiovisual speech processing in the sense that the visual cortex is able to extract phonological information from silent lip movements in the theta band (4–7 Hz). Crucially, this tracking is dependent on the natural statistics of silent speech, with absent tracking when the silent visual speech is presented reversed (and therefore not containing ordinary speech modulations). Another study supports the former findings and extends the present framework by providing evidence that the visual cortex passes information to the angular gyrus, which extracts slow features (below 1 Hz) from lip movements that are then mapped onto auditory features and passed on to auditory cortices for better speech comprehension (Bourguignon et al. 2020). These findings underline the importance of slow frequency properties of visual speech for enhanced speech comprehension from both the delta (0.5–3 Hz) and theta band (4–7 Hz), especially due to frequencies between 1 and 7 Hz being crucial for comprehension (Giraud and Poeppel 2012). Moreover, the spectral profile of lip movements is also settled within this range (Park et al. 2016).

Recent behavioral evidence describes that spectral fine details can also be extracted by observation of lip movements (Plass et al. 2020). This raises the interesting question whether this information is also represented at the level of the visual cortex analogous to the envelope as shown previously (Hauswald et al. 2018). Particularly relevant spectral fine details are formant peaks around 2500 Hz, which are indicated to be modulated in the front cavity (Badin et al. 1990). This corresponds to expansion and contraction of the lips (Plass et al. 2020), thus having a relationship with certain lip movements and could therefore be extracted for important phonological cues.

Furthermore, not only resonant frequencies but also the fundamental frequency (or pitch contour) plays an important role in understanding speech in noisy environments (Hopkins et al. 2008) and could potentially be extracted from silent lip movements. Whether the visual cortex is able to track formant and pitch information in (silent) visual speech has not been investigated to date.

Knowledge of how the brain processes speech is also vital with respect to aging, potentially connected with age-related hearing loss (Liberman 2017). Several studies have investigated the influence of age on speech comprehension with results that signify aging is, in most cases, accompanied by listening difficulties, especially in noise (Tun and Wingfield 1999; Wong et al. 2009). Furthermore, although the auditory tracking of a speech-paced stimulation (~3 Hz) is less consistent in older adults compared with younger adults, alpha oscillations are enhanced in younger adults during attentive listening, thus suggesting declining top-down inhibitory processes that support selective suppression of irrelevant information (Henry et al. 2017). Older adults also indicate a compensatory mechanism when processing degraded speech, especially in the anterior cingulate cortex (ACC) and middle frontal gyrus (Erb and Obleser 2013). Additionally, the temporal processing of auditory information is altered in the aging brain, pointing to decreased selectivity for temporal modulations in primary auditory areas (Erb et al. 2020). Those studies reinforce a distinctive age-related alteration in processing auditory speech. This raises the question whether we also see an impact of age on audiovisual speech processing, an issue that has not been addressed so far.

Combining the important topics mentioned above, this study aims to answer two critical questions regarding audiovisual speech processing: First, we ask if the postulated visuo-phonological transformation process in visual cortex mainly represents global energy modulations (i.e., speech envelope) or if it also entails spectral fine details (like formant or pitch curves). Second, we question if visuo-phonological transformation is subject to age-related decline. To the best of our knowledge, this study presents the first neurophysiological evidence that the visual cortex is not only able to extract the unheard speech envelope but also unheard formant and pitch information from lip movements. Crucially, we observed an age-related decline that mainly affects tracking of the formants (and to some extent the envelope and the fundamental frequency). Interestingly, we observed different tracking properties for different brain regions and frequencies: Although tracking natural unheard formants declines reliably in the occipital and cingulate cortex for both delta and theta, we observed a decline of theta-tracking just in the occipital cortex, which suggests different age-related effects in different brain regions. Our results suggest that the aging brain deteriorates in deriving spectral fine details linked to the visual input, a process that could contribute to perceptual difficulties in challenging listening situations.

## Materials and Methods

### Participants

We recruited 50 participants (28 female; 2 left-handed; mean age: 37.96 years; standard deviation (SD): 13.33 years, range: 19–63 years) for the experiment. All participants had normal or corrected-to-normal eyesight, self-reported normal hearing, and no neurological disorders. All participants were included in the coherence analysis. Forty-three of the 50 participants also completed a behavioral experiment investigating their lip-reading abilities beforehand. The remaining seven participants did not complete the behavioral experiment beforehand and could not be contacted afterwards. Four datasets had to be excluded because there were problems with the data acquisition and their answers were not saved, resulting in 39 behavioral datasets. All participants received either a reimbursement of €10 per hour or course credits for their participation. All participants signed an informed consent form. The experimental procedure was approved by the Ethics Committee of the University of Salzburg according to the Declaration of Helsinki.

### Stimuli

Videos were recorded with a digital camera (Sony NEX FS100) at a rate of 50 frames per second, and the corresponding audio files were recorded at a sampling rate of 48 kHz. The videos were spoken by two female native German speakers. The speakers were told to narrate the text with as little additional face and body expressions as possible to avoid influences from other facial gestures (as our main interest was the processing of the lip movements). We uploaded two example videos on our OSF-page (https://osf.io/ndvf6/). One speaker was then randomly chosen per subject and kept throughout the experiment, so each participant only saw one speaker. The stimuli were taken from the book “Das Wunder von Bern” (“The Miracle of Bern”; https://www.aktion-mensch.de/inklusion/bildung/bestellservice/materialsuche/detail?id=62), which was provided in an easy language. The easy language does not include any foreign words, has a coherent verbal structure, and is facile to understand. We used simple language to avoid limited linguistic knowledge from interfering with possible lip-reading abilities. Twenty-four pieces of text were chosen from the book and recorded from each speaker, lasting between 33 and 62 s, thus resulting in 24 videos. Additionally, all videos were reversed, which resulted in 24 forward videos and 24 corresponding backward videos. Forward and backward audio files were extracted from the videos and used for the data analysis. Half of the videos were randomly selected to be presented forward and the remaining half to be presented backward. The videos were back-projected on a translucent screen in the center of the screen by a Propixx DLP projector (VPixx Technologies, Canada) with a refresh rate of 120 Hz per second and a screen resolution of 1920 x 1080 pixels. The translucent screen was placed approximately 110 cm in front of the participant and had a screen diagonal of 74 cm.

### Procedure

Participants were first instructed to take part in an online study, in which their behavioral lip-reading abilities were tested and they were asked about their subjective hearing impairment. This German lip-reading test is available as Salzburg Lipreading Test (SaLT) (Suess et al. 2021). Participants were presented with silent videos of numbers, words, and sentences and could watch every video twice. They then had to write down the words they thought they had understood from the lip movements. This online test lasted approximately 45 min and could be conducted at home or right before the experiment in the MEG lab. After completing the behavioral experiment, the MEG experiment started. Participants were instructed to pay attention to the lip movements of the speakers and passively watch the muted videos. They were presented with six blocks of videos, and in each block, two forward and two backward videos were presented in a random order. The experiment lasted about an hour including preparation. The participants were not informed beforehand that part of the speech was reversed. After the session, they were asked if they had understood the story and were told that half of the videos were reversed. None of the participants reported to have understood the story or realized the flipping of the videos. The experimental procedure was programmed in MATLAB with the Psychtoolbox-3 (Brainard 1997; Pelli 1997; Kleiner et al. 2007) and an additional class-based abstraction layer (https://gitlab.com/thht/o_ptb) programmed on top of the Psychtoolbox (Hartmann and Weisz 2020).

### Data Acquisition

Brain activity was measured using a 306-channel whole head MEG system with 204 planar gradiometers and 102 magnetometers (Neuromag TRIUX, Elekta), a sampling rate of 1000 Hz and an online high-pass filter of 0.1 Hz. Before entering the magnetically shielded room (AK3B, Vacuumschmelze, Hanau, Germany), the head shape of each participant was acquired using approximately 500 digitized points on the scalp, including fiducials (nasion, left and right preauricular points) with a Polhemus Fastrak system (Polhemus, Colchester, VT). The head position of each participant relative to the MEG sensors was controlled once before each experimental block. Vertical and horizontal eye movements and electrocardiographic data were also recorded but not used for preprocessing. The continuous MEG data were then preprocessed offline with the signal space separation method from the Maxfilter software (Elekta Oy, Helsinki, Finland) to correct for different head positions across blocks and to suppress external interference (Taulu et al. 2005).

### Data Analysis

#### Preprocessing

The acquired datasets were analyzed using the Fieldtrip toolbox (Oostenveld et al. 2011). The max-filtered MEG data were high-pass–filtered at 1 Hz using a finite impulse response (FIR) filter (Kaiser window, order 440). For extracting physiological artifacts from the data, 60 principal components were calculated. Via visual inspection, the components displaying eye movements, heartbeat, and external power noise from the nearby train tracks (16.67 Hz) were removed from the data. We removed on average 2.24 components per participant (SD = 0.65). The data were then low-pass–filtered at 30 Hz and corrected for the delay between the stimulus computer and the screen inside the chamber (9 ms for each video). We then resampled the data to 150 Hz and segmented them in 2 s trials to increase the signal-to-noise ratio.

#### Source Projection of MEG Data

We used either a standard structural brain from the Montreal Neurological Institute (MNI, Montreal, Canada) or, where possible, the individual structural magnetic resonance imaging (MRI) (20 participants) and warped it to match the individual’s fiducials and head shape as accurately as possible (Mattout et al. 2007). A 3D grid with 1 cm resolution and 2982 voxels based on an MNI template brain was morphed into the brain volume of each participant. This allows group-level averaging and statistical analysis as all the grid points in the warped grid belong to the same brain region across subjects. These aligned brain volumes were also used for computing single-shell head models and lead fields (Nolte 2003). Using the lead fields and the common covariance matrix (pooling data from all blocks), a common LCMV beam-former spatial filter was computed (Veen et al. 1997).

#### Extraction of Lip Area, Acoustic Speech Envelope, Formants, and Pitch

**Figure 1:**
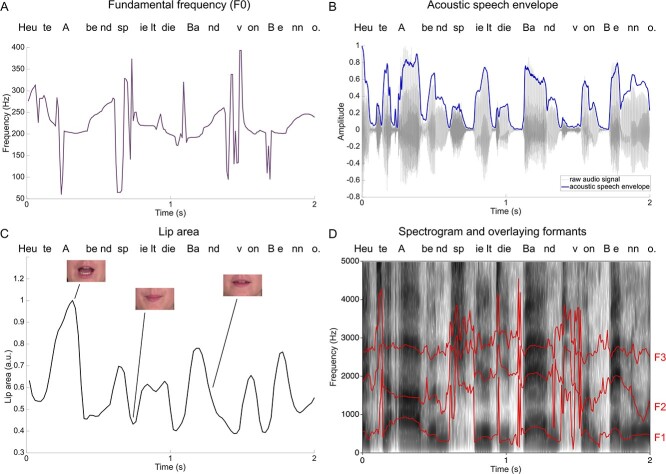
Example time series for a 2 s forward section of all the parameters used for coherence calculation. (*A*) Example time series of the fundamental frequency extracted with the pitch.m MATLAB function. (*B*) Example audio signal and the acoustic speech envelope (in blue). (*C*) Lip area extracted from the video frames with the MATLAB script adapted from Park et al. (2016). (*D*) Example spectrogram with overlaying formants (F1–F3, red lines) extracted with Praat.

The lip area of the visual speech was extracted using a MATLAB script adapted from Park et al. (2016). This data was then upsampled to 150 Hz to match the downsampled preprocessed MEG signal. The acoustic speech envelope was extracted with the Chimera toolbox from the audio files corresponding to the videos, which constructed nine frequency bands in the range of 100–10 000 Hz as equidistant on the cochlear map (Smith et al. 2002). The respective cut-off values for the nine frequency bands were as follows: 101, 220, 402, 680, 1103, 1748, 2732, 4231, 6517 and 10 000. Those values are based on the cochlear frequency maps for the cat scaled to fit the human frequency range of hearing (Liberman 1982). Then the sound stimuli were band-pass–filtered in these bands with a fourth-order Butterworth filter to avoid edge artifacts. For each of the frequency bands, the envelopes were calculated as absolute values of the Hilbert transform and then averaged to get the full-band envelope for coherence analysis (Gross et al. 2013; Keitel et al. 2017). This envelope was then downsampled to 150 Hz to match the preprocessed MEG signal. The resonant frequencies (or formants) were extracted using the Burg method implemented in Praat 6.0.48 (Boersma and Weenink 2019). Up to five formants were extracted from each audio file to make sure that the relevant formants were extracted thoroughly. For analysis purposes, just F2 and F3 were averaged and used. Those two formants fluctuate around 2500 Hz and tend to merge into a single peak when pronouncing certain consonant–vowel combinations (Badin et al. 1990). The mentioned merging process takes place in the front region of the oral cavity and can therefore also be seen by observing lip movements (Plass et al. 2020). The formants were extracted at a rate of 200 Hz for the sake of simplicity and then downsampled to 150 Hz. The pitch (or fundamental frequency, f0) was extracted using the MATLAB Audio Toolbox function pitch.m with default options (extraction between 50 and 400 Hz) at a rate of 100 Hz and then upsampled to 150 Hz (Fig. 1).

#### Coherence Calculation

We calculated the cross-spectral density between the lip area, the unheard acoustic speech envelope, the averaged F2 and F3 formants and the pitch and every virtual sensor with a multi-taper frequency transformation (1–25 Hz in 0.5 Hz steps, 3 Hz smoothing). Then we calculated the coherence between the activity of every virtual sensor and the lip area, the acoustic speech envelope, the averaged formant curve of F2 and F3, and the pitch curve, which we will refer to as lip–brain coherence, envelope–brain coherence, formant–brain coherence, and pitch–brain coherence, respectively, in this article. The overlap of all coherence values that reached at least 90% of the maximum coherence value for the respective speech feature was calculated and included in the main article. The grand average of the coherence values for the single speech features (separately for forward and backward speech) is included in the Supplementary Material.

### Statistical Analysis

To test for differences in source space in occipital cortex for forward (natural) and backward (reversed) coherence values, we extracted all voxels labeled as “occipital cortex” in the Automated Anatomical Labeling (AAL) atlas (Tzourio-Mazoyer et al. 2002) for a predefined region-of-interest analysis (Hauswald et al. 2018). To model the coherence values as a function of the covariates, we calculated a generalized linear model (GLM) with the package *lme4* (Bates et al. 2015) in R (R Core Team, 2021). The fixed effects were defined as “speech features” (continuous variable with four levels, namely lip–brain coherence, unheard speech envelope–brain coherence, unheard formant–brain coherence, and unheard pitch–brain coherence) and “naturalness of speech” (categorical with two levels, namely, natural and reversed). To account for the dependency between observations over participants, we modeled responses by the same person with varying intercepts. To further investigate the main effects, we then calculated the Tukey contrasts using the package multcomp (Hothorn et al. 2008) with FDR correction. Furthermore, we were interested if the coherence values of the naturalness of speech differ within the speech features (i.e., difference between coherence values for natural speech and reversed speech for the single speech features), so we calculated a GLM with the fixed effect “condition” (continuous variable with eight levels, which uses the combined factors from our first model) and again included random intercepts to account for the dependency between observations across subjects. We then calculated planned contrasts between the naturalness of speech within the speech features for our frequency band of interest (1–7 Hz) with FDR correction again.

In a first step, we decided to average over the delta (1–3 Hz) and theta (4–7 Hz) frequency bands as they carry important information in general on speech processing (phrasal and syllabic processing, respectively) (Giraud and Poeppel 2012). Moreover, previous studies investigated lip movement–related activity either in the delta band (Park et al. 2016; Bourguignon et al. 2020) or the theta band (Hauswald et al. 2018), leading us to also do a follow-up analysis separately for the different frequency bands (described later in this section).

To generate a normalized contrast between processing of forward (natural) and backward (reversed) lip movements, we subtracted the backward coherence values from the forward coherence values for our respective speech features (lip–brain coherence, unheard speech envelope–brain coherence, unheard formant–brain coherence, and unheard pitch–brain coherence). From now on, we refer to this normalized contrast as “intelligibility index” (as we assume that forward speech is more intelligible than backward speech), which quantifies the differences in coherence between natural and reversed speech features.

For testing the relationship between the four different intelligibility indices (lip–brain, envelope–brain, formant–brain, and pitch-brain) and age, we conducted a voxel-wise correlation with age. To control for multiple comparisons, we used a nonparametric cluster-based permutation test (Maris and Oostenveld 2007). Here, clusters of correlation coefficients being significantly different from zero (showing *P* values <0.05) were identified and their respective *t*-values were extracted and summed up to get a cluster-level test statistic. Random permutations of the data were then drawn by reordering the behavioral data (in our case age) across participants. After each permutation, the maximum cluster level *t*-value was recorded, generating a reference distribution of cluster-level *t*-values (using a Monte Carlo procedure with 1000 permutations). Cluster *P* values were estimated as the proportion of cluster *t*-values in the reference distribution exceeding the cluster *t*-values observed in the actual data. Significant voxels (which were only found in the correlation between the formant–brain index and age) were then extracted and averaged for data-driven regions of interest (ROIs) (occipital cortex and cingulate cortex), which were defined using the Automated Anatomical Labeling (AAL) atlas (Tzourio-Mazoyer et al. 2002). These data-driven ROIs were then applied to all intelligibility indices to make the ROI analysis comparable. We then fitted four linear models using the lm function from the statistical package in R (R Core Team 2021) to investigate if age could predict the change in the calculated intelligibility indices and to visualize the statistical effects of the whole-brain analysis. The last step consisted of a follow-up analysis where we decided to separate the averaged frequency bands (delta and theta) again to unravel possible differences of our effect depending on the frequency band. We again conducted a voxel-wise correlation with age separately for the delta band (1–3 Hz) and for the theta band (4–7 Hz) with the already described nonparametric cluster-based permutation test for all described intelligibility indices. Finally, we extracted the values from the voxel with the lowest *t*-value (for the delta and theta bands, respectively) and fitted a linear model again to investigate if age could predict the change in the intelligibility indices and to visualize the statistical effects of the whole-brain analysis.

## Results

### Behavioral Results

**Figure 2:**
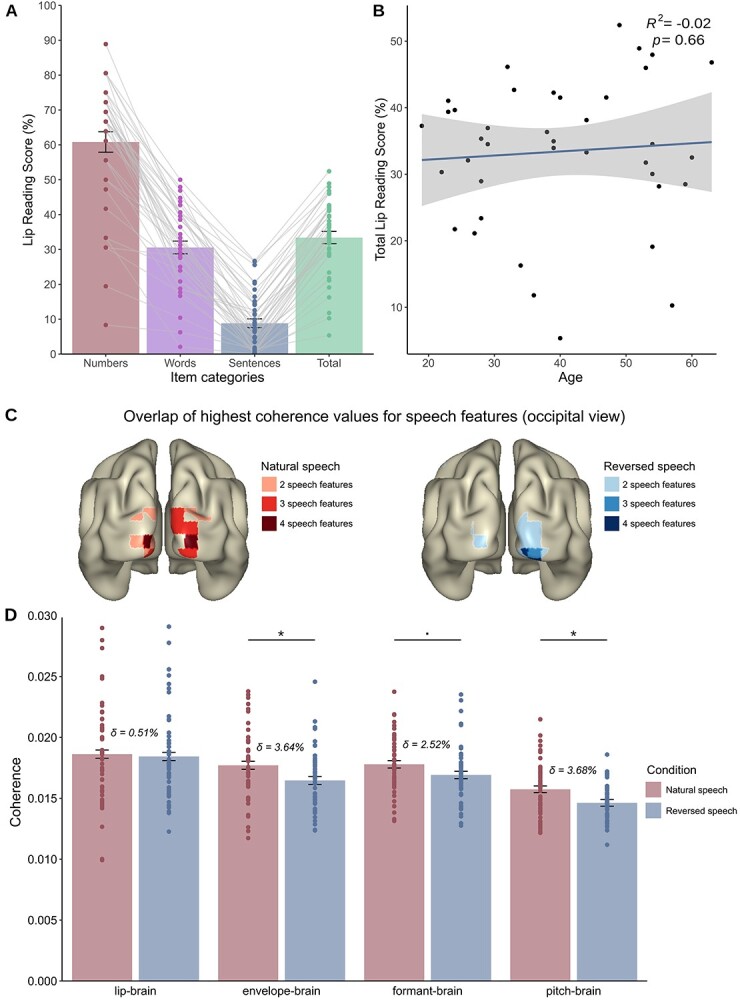
Behavioral data and comparison of information tracking in visual cortex. (*A*) Behavioral lip-reading abilities. Participants recognized numbers the most, followed by words and sentences. (*B*) Correlation between age and lip-reading score revealed no significant correlation (*P* = 0.66), suggesting that lip-reading abilities do not change with age. The blue line depicts regression line, and shaded areas depict standard error of mean (SE). (*C*) Overlap of voxels reaching at least 90% of the maximum coherence value for the respective speech feature. Occipital areas show a strong overlap of highest coherence values over speech features. (*D*) Mean values extracted from all voxels in occipital cortex showing no significant differences in lip–brain coherence (*P*_FDR_ = 0.695) but showing significant differences in unheard envelope-brain coherence (*P*_FDR_ = 0.041) and unheard pitch–brain coherence (*P*_FDR_ = 0.042) and a trend for the formant–brain coherence (*P*_FDR_ = 0.095) between natural and reversed presentation of visual speech. Error bars represent 1 SE for within-subject designs (O’Brien and Cousineau 2014), and δ indicates the relative change between natural and reversed conditions in percent.

We investigated participants’ lip-reading abilities in a separate experiment that was conducted before the MEG session. They were presented with silent videos of spoken numbers, words, and sentences, and the task was to write down what they had understood just from the lip movements alone. A detailed description of the behavioral task will be published in a separate paper (Suess et al. 2021). Thirty-nine of the 50 participants completed the behavioral experiment. Although the recognition rate for the numbers was high (*M* = 60.83%, SE = 2.93%), lip-reading abilities for complex stimuli (words and sentences) were low in general (words: *M* = 30.57%, SE = 1.82%; sentences: *M* = 8.83%, SE = 1.26%). Participants had an average total score of 33.41% (SE = 1.75%, Fig. 2*A*). Investigating if age could predict the lip-reading score revealed that those two variables were uncorrelated (*F*(1, 37) = 0.191, *P* = 0.664, *R*^2^ = −0.021), Fig. 2*B*), showing that in our sample behavioral lip-reading abilities did not change with age. This is consistent with our study on general lip-reading abilities in the German language (Suess et al. 2021) but different to other studies indicating higher lip-reading abilities in younger individuals (Tye-Murray et al. 2007a; Feld and Sommers 2009). Participants also completed a questionnaire on subjective hearing impairment (APHAB, Löhler et al. (2014)). Further investigating the relationship between subjective hearing impairment and lip-reading score also revealed no significant effect (*F*(1, 37) = 0.104, *P* = 0.75, *R*^2^ = −0.024) in the current sample. This is in line with studies investigating hearing impairment in older adults (Tye-Murray et al. 2007b) but does not support our own results that show a relationship between self-reported hearing impairment and lip-reading abilities (Suess et al. 2021). However, as the current study aimed to test normal hearing individuals with restricted variance in hearing impairment, those results cannot be compared directly with those of Suess et al. (2021), which also included individuals with severe hearing loss as well as prelingually deaf individuals.

### Visuo-phonological Transformation Is Carried by Both Tracking of Global Envelope and Spectral Fine Details During Presentation of Natural Silent Lip Movements

We calculated the coherence between the MEG data and the lip envelope, the unheard acoustic speech envelope, the unheard resonant frequencies, and the unheard pitch (from now on called lip–brain coherence, envelope–brain coherence, formant–brain coherence, and pitch–brain coherence, respectively). As the visuo-phonological transformation process likely takes place in visual areas (Hauswald et al. 2018), we defined the occipital cortex using the AAL atlas (Tzourio-Mazoyer et al. 2002) as a predefined ROI and averaged over all voxels from this ROI for statistical comparisons. To also visualize where coherence for speech features in general is the highest, we extracted the voxels that reached at least 90% of the maximum coherence value for the respective speech feature. Occipital areas show a strong overlap of highest coherence values over speech features, and this is even more pronounced in natural speech (Fig. 2*C*). This shows that the visual cortex is primarily involved in tracking speech features extracted from pure visual speech. Figures showing maximum coherence values split up by speech features are found in Supplementary Figures S1–S4.

In a first step, we calculated a GLM with the fixed factors speech features and naturalness of speech. We found a significant difference for the factor speech features (*F*(3, 346) = 32.778, *P* <0.001) and naturalness of speech (*F*(1, 346) = 12.524, *P* = 0.0004; Supplementary Table S1). Investigating the contrasts using Tukey’s honest significant difference (HSD) test revealed that all effect levels of the speech features differed significantly from each other (all *P*_FDR_ <0.0007) except for the levels envelope–brain coherence and formant brain–coherence (*P*_FDR_ = 0.441, Supplementary Table S2). This shows that the speech features are tracked differently by the brain except for the speech envelope and the formant modulations, which do not differ significantly.

To also identify if the coherence values of the naturalness of speech (natural vs. reversed) differ within the speech features, we calculated a GLM for the factor condition (connecting the factors used in our first model, resulting in a variable with eight levels). Using predefined contrasts (Tukey HSD test) revealed that although there was no difference in the lip–brain coherence for natural and reversed visual speech (*Z* = 1.805, *P* = 0.695), we found a significant difference in unheard envelope–brain coherence for natural and reversed visual speech (*Z* = 2.571, *P* = 0.041). Most importantly, we found a significant difference also for the unheard pitch–brain coherence (*Z* = 2.308, *P* = 0.042) and a trend for the unheard formant–brain coherence for natural and reversed visual speech (*Z* = 1.805, *P* = 0.095, all in Fig. 2*D*). The results on the tracking of lip movements are in line with former findings, showing that the visual cortex tracks these regardless of proximity to natural speech but point to different tracking properties depending on the naturalness of the unheard speech envelope. Interestingly, we show here that the visual cortex is also able to distinguish (to a certain extent) between unheard normal and reversed formants (or resonant frequencies) and pitch (or F0) modulations extracted from the spectrogram, thus showing also that unheard natural spectral details are extracted from visual speech and represented at the level of the visual cortex.

### Spectral Fine Detail Tracking Rather than Global Envelope Tracking Is Altered in Aging Population

**Figure 3:**
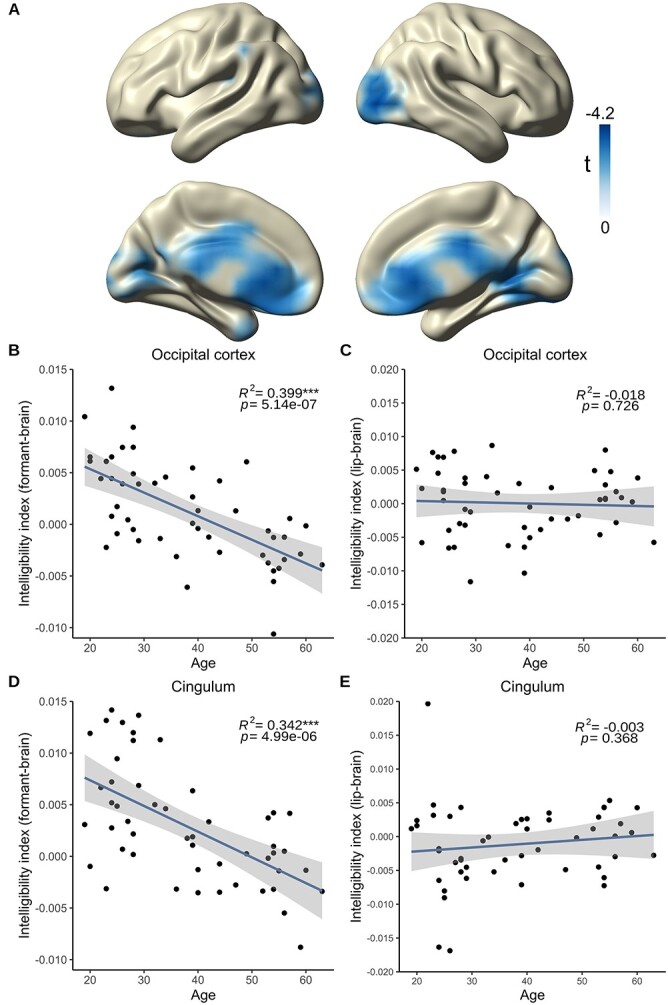
Correlation between age and intelligibility index (i.e., difference in tracking of natural and reversed speech) and comparison of age groups. (*A*) Statistical values of the voxel-wise correlation of the formant–brain intelligibility index with age (averaged over 1–7 Hz, *P* <0.05, cluster corrected) showing a strong decrease of intelligibility tracking in occipital regions and in cingulate cortex. (*B*) Correlation of formant–brain intelligibility index in significant occipital voxels extracted from a showing a significant correlation with age (*P* = 5.14e−07). (*C*) Correlation of lip–brain intelligibility index in significant occipital voxels extracted from a showing a not-significant correlation with age (*P* = 0.726). (*D*) Correlation of formant–brain intelligibility index in significant voxels from the cingulate cortex extracted from a showing a significant correlation with age (*P* = 4.99e−06). (*E*) Correlation of lip–brain intelligibility index in significant voxels from the cingulate cortex extracted from a showing a not-significant correlation with age (*P* = 0.368). Blue lines depict regression lines, and shaded areas depict SE.

We were then interested in how the visuo-phonological transformation process is influenced by age. So we calculated a voxel-wise correlation between the intelligibility index (difference between coherence for forward videos and coherence for backward videos) separately for our coherence indices (lip–brain, envelope–brain, formant–brain, and pitch–brain) and the age of the participants. We neither found a significant correlation between the intelligibility index of the lip–brain coherence and age (*P* = 1, cluster corrected) nor between the intelligibility index of the unheard envelope–brain coherence and age (*P* = 0.09, cluster corrected). Moreover, the correlation between the intelligibility index of the unheard pitch–brain coherence was statistically not significant (*P* = 0.07, cluster corrected). However, the overall trend for the envelope–brain and the pitch–brain coherence was to decline with age. Interestingly, we did find a significant negative correlation between the intelligibility index of the unheard formant–brain coherence and age (*P* = 0.002, cluster corrected), strongest in the occipital cortex and cingulate cortex (lowest *t*-value: –4.124, MNI [40 –90 0], Fig. 3*A*). To further investigate the effects, we extracted the voxels showing a statistical effect in our whole-brain analysis (Fig. 3*A*) and divided them into occipital voxels and voxels from the cingulate cortex using the AAL atlas (Tzourio-Mazoyer et al. 2002).

To investigate how strong the relationship between age and the different intelligibility indices was in our ROIs, we fitted four separate linear models. We started with the lip–brain index to exclude the possibility that our effect is due to visual processing. We found that age could not predict the lip–brain intelligibility index in any of the chosen ROIs (occipital cortex: *F*(1, 48) = 0.124, *P* = 0.727, η^2^ = 0.002, Fig. 3*C*; cingulate cortex: *F*(1, 48) = 0.825, *P* = 0.368, η^2^ = 0.017, Fig. 3*E*). In contrast, we found that age could significantly predict the decrease in the formant–brain intelligibility index in both occipital areas (*F*(1, 48) = 33.59, *P* = 5.14e−07, η^2^ = 0.412, Fig. 3*B*) and cingulate cortex (*F*(1, 48) = 26.42, *P* = 4.99e−06, η^2^ = 0.355, Fig. 3*D*) suggesting an altered tracking process for the formants in aging. To unravel how these effects come around, we reassessed the formant–brain coherence values separately for natural (forward) and reversed (backward) speech with respect to the age of our participants (see Supplementary Material, Supplementary Fig. S5). As already seen in the formant–brain index, which shows a tendency to cluster around zero or show even negative values with increasing age, our additional analysis revealed that tracking of natural speech is enhanced in the younger population whereas the aging brain fails to distinguish between natural and reversed speech, and even shows a tendency to track the presented reversed speech more than the young group.

Further fitting linear models to investigate the effects in our ROIs for the envelope–brain coherence and the pitch–brain coherence revealed that age could not significantly predict the envelope–brain index in the occipital (*F*(1, 48) = 1.638, *P* = 0.207, η^2^ *=* 0.033) or cingulate cortex (*F*(1, 48) = 0.681, *P* = 0.413, η^2^ = 0.014) and also not the pitch–brain index in the occipital cortex (*F*(1, 48) = 2.584, *P* = 0.114, η^2^ *=* 0.051). However, age could significantly predict the pitch–brain index in the cingulate cortex (*F*(1, 48) = 6.972, *P* = 0.011, η^2^ *=* 0.127). The lack of tracking differences between natural and reversed lip movements suggests that the visual cortex processes basic visual properties of lip movements but that there are differential processing strategies for acoustic information associated with these lip movements. These results also suggest that processing of the pitch (or fundamental frequency) is altered to some extent in the aging population, at least in the cingulate cortex. In summary, the correlations between the envelope–brain index and age and the pitch–brain index and age seem to show a tendency in line with the relationship between the formant–brain index and age in the whole-brain analysis. We see that effect sizes are biggest for the formant–brain index (occipital η^2^ *=* 0.412, cingulate η^2^ = 0.355), followed by the pitch–brain index (occipital η^2^ = 0.051, cingulate η^2^ = 0.127). Lower effect sizes are found for the envelope–brain index (occipital η^2^ = 0.033, cingulate η^2^ *=* 0.014) and the lip–brain index (occipital η^2^ *=* 0.002, cingulate η^2^ *=* 0.017) after extracting voxels from the data-driven ROI, adding to the evidence of a differential processing of speech properties in age.

### Different Frequency Bands Show Age-Related Decline in Different Brain Regions

**Figure 4:**
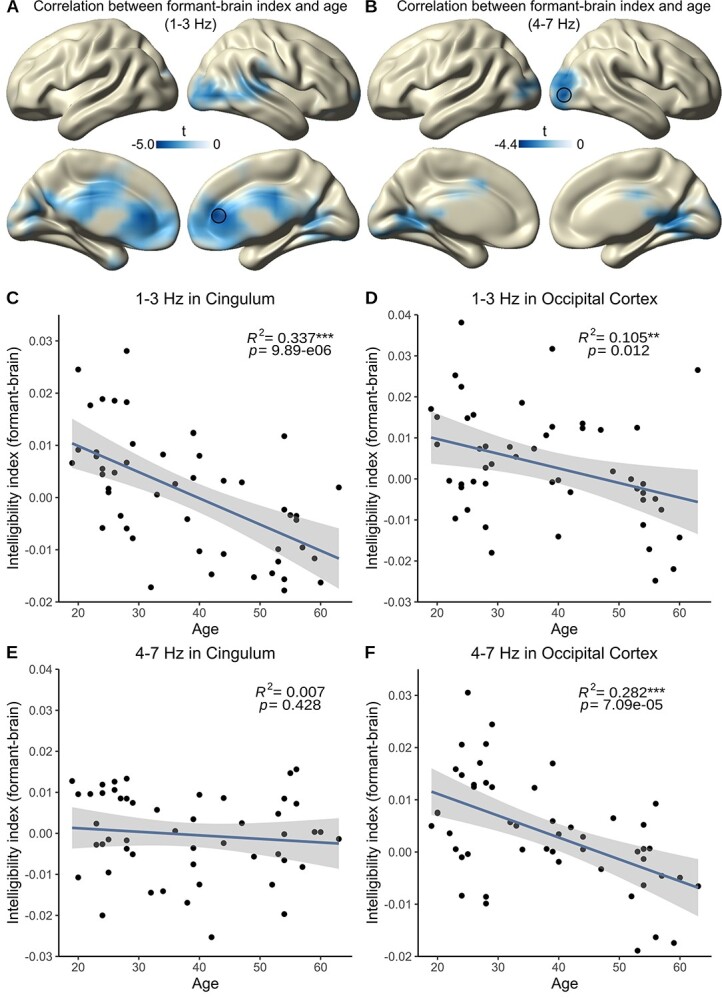
Statistical values of the voxel-wise correlation of the formant–brain index with age split between delta band and theta band. (*A*) Tracking of the intelligibility index in the delta band (1–3 Hz, *P* <0.05, cluster corrected) indicates a strong decrease of speech tracking in the cingulate cortex and frontal areas. The black circle indicates lowest *t*-value extracted for *C* and *E*. (*B*) Tracking of the intelligibility index in the theta band (4–7 Hz, *P* <0.05, cluster corrected) indicates a strong decrease of speech tracking in visual areas. The black circle indicates lowest *t*-value extracted for *D* and *F*. (*C*) Correlation of formant–brain intelligibility index in the voxel with the lowest *t*-value extracted from *A* (cingulate cortex) showing a significant decrease with age (*P* = 9.885e−06) in the delta band. (*D*) Correlation of formant–brain intelligibility index in the voxel with the lowest *t*-value extracted from *B* (occipital cortex) showing a significant decrease with age (*P* = 0.012) also in the delta band. (*E*) Correlation of formant–brain intelligibility index in the voxel with the lowest *t*-value extracted from *A* (cingulate cortex) showing no significant decrease with age (*P* = 0.428) in the theta band. (*F*) Correlation of formant–brain intelligibility index in the voxel with the lowest *t*-value extracted from *B* (visual cortex) showing a significant decrease with age (*P* = 7.089e−05) in the theta band. Blue lines depict regression lines, and shaded areas depict SE.

As a last step, we investigated whether different frequency bands are impacted differently by age-related decline. Therefore, we repeated the analysis steps explained in the preceding section, meaning that we calculated again a voxel-wise correlation between the intelligibility index separately for our coherence conditions (lip–brain, envelope–brain, formant–brain, and pitch–brain) and the age of the participants, but this time separately for the delta band (1–3 Hz) and the theta band (4–7 Hz). For the delta band, we again found a significant correlation between age and the intelligibility index just for the formant–brain index (*P* = 0.002, cluster corrected). This effect was strongest in cingulate cortex (lowest *t*-value: –4.991, MNI [0 40 10], Fig. 4*A*). No correlation was observed between age and the other indices (lip–brain index: *P* = 0.833; envelope–brain index: *P* = 0.268; pitch–brain index: *P* = 0.166, all cluster corrected). Repeating the analysis for the theta band revealed a similar picture: Although we could find a significant correlation between the formant–brain index and age (*P* = 0.018, cluster corrected), which was the strongest in the visual cortex (lowest *t*-value: –4.394, MNI [40 –90 0], Fig. 4*B*), we did not find it for the remaining indices and age (lip–brain index: *P* = 1; envelope–brain index: *P* = 0.096; pitch–brain index: *P* = 0.675, all cluster corrected). These results display a differential spatial pattern for different frequency bands: Although tracking of speech in the theta band declines reliably in the visual cortex, tracking of the slower delta band declines in the cingulate cortex and frontal areas. We then extracted the values from the voxel with the lowest *t*-value (i.e., the most significant negative one), respectively, for both frequency bands (delta band: cingulate cortex, MNI [0 40 10]; theta band: visual cortex, MNI [40 –90 0]) and again fitted a linear model for the formant–brain index to further clarify the effects found in the whole-brain analysis. Age could significantly predict the formant–brain index in the delta band in the cingulate cortex (*F*(1, 48) = 24.4, *P* = 9.885e−06, η^2^ = 0.337, Fig. 4*C*) and in the theta band in the visual cortex (*F*(1, 48) = 18.92, *P* = 7.089e−05, η^2^ = 0.282, Fig. 4*F*). To further clarify whether the tested relationship is specific to a certain frequency band and brain region, we also tested the vice versa relationship (i.e., the relationship between age and theta band in the cingulate cortex and the relationship between age and delta band in the occipital cortex). We found that although age could not significantly predict the formant–brain index in the theta band in the cingulate cortex (*F*(1, 48) = 0.637, *P* = 0.429, η^2^ = 0.01, Fig. 4*E*), it could significantly predict the formant–brain index in the delta band in the occipital cortex (*F*(1, 48) = 6.757, *P* = 0.012, η^2^ = 0.123, Fig. 4*D*). This suggests that although the ability of the cingulate cortex to transform visual into phonological information declines just in the delta band, the occipital cortex shows a decline over a broad range of frequencies and, therefore, in general visual speech processing.

## Discussion

Our study illustrates that during lip-reading, the visual cortex represents multiple features of the speech signal in low frequency bands (1–7 Hz), importantly including the corresponding (unheard) acoustic signal. It has previously been shown that the visual cortex is able to track the intelligible global envelope (unheard acoustic speech envelope; Hauswald et al. 2018). We demonstrate here that the visual cortex is also able to track the modulation of natural spectral fine details (unheard formants and pitch) in contrast to reversed speech which does not carry natural properties of speech. Furthermore, we found that aging is associated with a deterioration of this ability not only in the visual cortex but also in the cingulate cortex. Disentangling delta and theta bands revealed that although the age-related decline of formant tracking is independent of frequency bands in the visual cortex, it is unique in the cingulate cortex for the delta band. Our results suggest that visuo-phonological transformation processes are sensitive to age-related decline, in particular with regard to the modulation of unheard spectral fine details.

### Visuo-phonological Transformation Processes Are Observable for Global Amplitude Modulations and Spectral Fine Detail Modulations

As expected, the current study replicates the main finding from Hauswald et al. (2018) showing a visuo-phonological transformation process in the visual cortex for the unheard speech envelope in an Italian speaking sample. Our study using a German speaking sample suggests that the postulated visuo-phonological transformation process at the level of the visual cortex is generalizable across languages. This is unsurprising as it is in line with studies on the speech envelope spectrum that show robust amplitude peaks between 3.5 and 4.5 Hz regardless of language (Poeppel and Assaneo 2020), providing evidence that different languages carry similar temporal regularities not only for auditory properties but also for visual properties (Chandrasekaran et al. 2009). We argue that this similarity is a key property for making the postulated visuo-phonological transformation process transferable to other languages.

By investigating different acoustic properties of visual speech (global modulations vs. fine-detailed modulations) and how they are tracked by the human brain, our results furthermore add an important part to the understanding of how visual speech contributes to speech processing in general. As lip movements and amplitude modulations are highly correlated (Chandrasekaran et al. 2009), it is very probable that amplitude modulations can be inferred by lip movements alone as a learned association.

Here we can show that the brain is also able to perform a more fine–coarse tracking than initially thought by especially processing the spectral fine details that are modulated near the lips, another potentially learned association between lip–near auditory cues (i.e. merged F2 and F3 formants) and lip movements (Plass et al. 2020). Although the formant modulations in the visual cortex only show effects on a trend level, we argue that this effect is due to the fact that the participants older than 35 show reduced capacities to distinguish between natural and reversed unheard formants accompanying lip movements (a result that will be discussed in detail later).

Additionally, it is not only formants that are subject to a visuo-phonological transformation but also the fundamental frequency as seen in our results. This is in line with a recent study that shows that closing the lips is correlated with the tone falling (Garg et al. 2019).

It is worth mentioning that using reversed acoustic speech as a control condition has been criticized (Brown et al. 2012) but remains still widely used (see e.g., Gross et al. 2013; Di Liberto et al. 2015; Broderick et al. 2018; Keitel et al. 2018 for auditory stimulation and Hauswald et al. 2018 for visual stimulation). We also argue that there is a crucial difference between auditory and visual reversed speech: If participants are not explicitly instructed, they are not aware that the visual speech is reversed when the auditory input is missing. Thus, by using reversed visual speech as a control condition, we try to ensure that our results are due to the preserved natural statistics and physical properties of speech in comparison with reversed speech. This notion is also supported by the fact that tracking of lip movements is not dependent on the naturalness of speech (as we see no difference between tracking of natural and reversed lip movements, Fig. 3*C*,*E*).

Our results here point to a helpful bottom-up process for understanding speech under suboptimal conditions. An alternative to this interpretation is that successful lip-reading leads to a cortical speech representation, which includes spectral information. Some studies suggest that enhanced lip-reading abilities go in line with higher activation in visual areas in persons with a cochlear implant (e.g. Giraud et al. 2001). This could mean that linguistic representations modulate the tracking of unheard acoustic information in a top-down manner. However, we did not find a relationship between behavior and tracking of unheard formants, thus suggesting that the tracking of unheard acoustic information accompanying lip movements is likely not a result of internal representations after successful lipreading but rather a bottom-up support process for enhanced speech understanding. Our present results therefore support the notion that visuo-phonological transformation processes taking place in the visual cortex are a fundamental part of watching visual speech. Thus, those processes may be most useful in disambiguating auditory signals in difficult listening situations, an idea to be investigated in detail in upcoming studies.

### Tracking of Unheard Formants Accompanying Lip Movements Is Mostly Affected in Aging

With regard to the aging effect, we could show that various neural tracking mechanisms are differentially affected. Our study presents that tracking of unheard formants, especially the combined F2 and F3 formants, declines with age although the tracking of purely visual information is still preserved (as seen in the lip–brain index, Fig. 3*C*,*E*). Meanwhile, the tracking of the unheard speech envelope and pitch signify an inconclusive picture: Although the tracking of those properties seems to be preserved to some extent, both show a tendency to diminish with age.

In particular, the formants and the pitch are part of the temporal fine structure (TFS) of speech and are crucial for speech segregation or perceptual grouping for optimal speech processing in complex situations (Bregman et al. 1990; Alain et al. 2017). The TFS is different from the acoustic envelope in a sense that it does not display “coarse” amplitude modulations of the audio signal but rather fluctuations that are close to the central frequency of certain frequency bands (Lorenzi et al. 2006). Hearing-impaired older participants show a relative deficit of the representation of the TFS compared with the acoustic envelope (Lorenzi et al. 2006; Anderson et al. 2013). The TFS also yields information when trying to interpret speech in fluctuating background noise (Moore 2008). Other studies also point to the fact that especially when having cochlear hearing loss along with a normal audiometric threshold, the processing of the TFS is reduced, resulting in diminished speech perception under noisy conditions (Lorenzi et al. 2009). This suggests that hearing-impaired subjects mainly seem to rely on the temporal envelope to interpret auditory information (Moore and Moore 2003) whereas normal hearing subjects can also use the presented temporal fine structure. Interestingly, we found that even when the TFS is inferred from lip movements, there is a decline in the processing of spectral fine details with age independent of hearing loss. Our results suggest that the visuo-phonological transformation of certain spectral fine details like the formants are impacted the most in aging whereas the transformation of the pitch (or fundamental frequency) reveals a more complex picture: We find preserved tracking of the unheard pitch contour in the occipital cortex but declining with age in the cingulate cortex. Interestingly, the cingulate cortex has been found to show higher activation in response to processing of degraded speech (Erb and Obleser 2013) pointing to a possible compensatory mechanism when processing distorted speech. The cingulate cortex (or the cingulo-opercular network) is indeed considered important in general speech processing and especially in challenging listening situations (Peelle 2018). Thus our results suggest that the mentioned network not only supports auditory speech processing but also visual speech processing. How this altered processing of the unheard pitch (or fundamental frequency) accompanying lip movements in the cingulate cortex has an impact on speech understanding needs to be investigated in future studies.

Separately correlating cortical tracking of natural and reversed speech (in contrast to calculating an “index”) with age revealed that the correlation of age and the index is mainly carried by the natural (forward) condition, for which younger people show stronger tracking compared with older people. For the reversed (backward) condition, there is no correlation with age. Taken together, these correlations suggest that older participants seem to be less able to distinguish between natural and reversed unheard formants whereas younger individuals show enhanced tracking of natural (forward) speech compared with older individuals (Supplementary Fig. S5).

This could point to the fact that the older population is losing the gain of processing unheard acoustic information that go along with natural speech obviously resulting in a less successful visuo-phonological transformation process. Other studies suggest that the older population seems to inefficiently use their cognitive resources showing less deterioration of cortical responses (measured by the envelope reconstruction accuracy) to a foreign language compared with younger individuals (Presacco et al. 2016a) and also an association between cognitive decline and increased cortical envelope tracking or even higher synchronization of theta (Goossens et al. 2016). Auditory processing is also affected both in the midbrain and cortex in age, exhibiting a large reduction of speech envelope encoding when presented with a competing talker, but at the same time a cortical overrepresentation of speech regardless of the presented noise, suggesting an imbalance between inhibition and excitation in the human brain (Presacco et al. 2016b) when processing speech. Other studies add to this hypothesis by showing decreasing alpha modulation in the aging population (Vaden et al. 2012; Henry et al. 2017) strengthening the assumption that there is an altered interaction between age and cortical tracking even in the visual modality that needs to be investigated further.

Considering all acoustic details accompanying lip movements, we still see a tendency of the speech envelope tracking to decline with age suggesting that the transformation of the global speech dynamics could also be deteriorating. Overall, our results provide evidence that the transformation of fine-grained acoustic details seem to decline more reliably with age whereas the transformation of global information (in our case the speech envelope) seems to be less impaired.

### Possible Implications for Speech Processing in Challenging Situations

Our findings raise the question of how the decline in processing of unheard spectral fine details negatively influences other relevant aspects of hearing. In the light of aforementioned studies from the auditory domain of speech processing, we propose some thoughts on the multisensory nature of speech and how different sensory modalities can contribute to speech processing abilities under disadvantageous conditions (both intrapersonal and environmental).

As mentioned under Tracking of Unheard Formants Accompanying Lip Movements, optimal hearing requires processing of both the temporal fine structure and the global acoustic envelope. However, especially under noisy conditions, processing the TFS becomes increasingly important for understanding speech. Aging in general goes along with reduced processing of the TFS (Anderson and Karawani 2020), and this deteriorating effect seems to be even more detrimental when aging is accompanied by hearing loss (Anderson et al. 2013). As listening in natural situations usually is a multisensory (audiovisual) phenomenon, we argue that the impaired visuo-phonological transformation process of the TFS adds to the difficulties of older (also audiometrically normal hearing) individuals to follow speech in challenging situations. To follow up this idea, future studies will need to quantify the benefit of audiovisual versus (unimodal) auditory processing.

Our results also have implications for listening situations when relevant visual input from the mouth area is obscured, a topic which has gained enormously in significance due to the wide adoption of face masks to counteract the spread of SARS-CoV-2. In general, listening becomes more difficult and performance declines when the mouth area is obscured (Brown et al. 2021; Giovanelli et al. 2021). As face masks may diminish attentional focusing as well as temporal cues, our work suggests that they also deprive the brain of deriving the acoustic TFS from the lip movements especially in the formant frequency range, which are modulated near the lips (F2 and F3). This issue, which should become relevant particularly in noisy situations, may be aggravated by the fact that face masks (especially highly protective ones) impact sound propagation of frequencies between 1600 and 6000 Hz with a peak around 2000 Hz (Caniato et al. 2021). Thus, face masks diminish relevant formant information in both sensory modalities. This could disproportionately affect hearing impaired listeners, an urgent question that should be followed up by future studies.

Overall, considering both the auditory and visual domain of speech properties, we suggest that the underlying cause of speech processing difficulties in naturalistic settings accompanying age or hearing impairment is more diverse than previously thought. The visual system provides the proposed visuo-phonological transformation process as an important bottom-up mechanism for optimal speech understanding and crucially supports acoustic speech processing.

### Occipital Cortex and Cingulate Cortex Show Different Tracking Properties Dependent on the Frequency Band

With regard to different frequency bands, our results could yield important insights into different brain regions showing distinct formant tracking properties: Although we find a robust decline of delta band tracking with age in both occipital and cingulate cortex, theta band tracking reliably declines only in the occipital areas. In general, theta corresponds to the frequency of syllables and to the modulations in the amplitude envelope (Gross et al. 2013; Keitel et al. 2018; Meyer 2018; Poeppel and Assaneo 2020) whereas delta seems to process phrasal chunks based on acoustic cues (Ghitza 2017; Keitel et al. 2018) and is therefore responsible for a general perceptual chunking mechanism (Boucher et al. 2019). So, the decreased processing of unheard formants in visual cortex in both frequency bands could point to a general decline of speech-related information processing. Extending the results from the previous behavioral study (Plass et al. 2020), we argue that visual speech provides not only (sub-)phonemic but also phrasal spectral information in a bottom-up manner to complete auditory cues for facilitated speech comprehension. This process might be disrupted with increasing age. Regarding the cingulate cortex, we find declined tracking of unheard formants in the delta band but preserved theta-band tracking. As older participants rely more on the speech envelope (Moore and Moore 2003), our results go in line with the mentioned compensatory mechanism (higher activation when presenting degraded speech) in the anterior cingulate cortex (ACC) (Erb and Obleser 2013) as the acoustic envelope shows amplitude modulations within the theta band (Gross et al. 2013). But this dissociation between delta and theta band tracking in the cingulate cortex also raises some questions: If the visual cortex provides purely bottom-up information about the visual stimulation, why do we see preserved tracking of unheard formants in the theta band in the cingulate cortex? This could point to a possible preserved top-down influence of stored syntactic information provided by delta band tracking and should be explored in future studies. Furthermore, to fully understand the mechanisms behind this visuo-phonological transformation process without the influence of aging in distinct brain regions and frequency bands, it would be advisable for future studies to focus on younger individuals, especially as this study is the first to investigate the tracking of spectral fine details extracted from the spectrogram.

The current study demonstrates that the visual cortex is able to track natural unheard spectral fine-detailed information just by observing lip movements. Crucially, we present a differential pattern for the processing of global (i.e., envelope) and spectral fine-detailed information with aging affecting in particular tracking of spectral speech information (or the TFS) while showing partly preserved tracking of global modulations. Furthermore, we see a distinct age-related decline of tracking depending on the brain region (i.e., visual and cingulate cortex) and on the frequency band (i.e., delta and theta band). The results presented here may have important implications for hearing in the aging population suggesting that hearing difficulties could also be exacerbated in natural audiovisual environments as a result of reduced capacities of visual benefit. With respect to the current pandemic situation, our results can provide a novel, important insight on how missing visual input (e.g., when wearing face masks) is critical for speech comprehension.

## Supplementary Material

Supplementary_material_cercor_bhab518

## Data Availability

The “mat” and “csv” files containing the data shown in the figures, along with the MATLAB code and the R code to recreate the plots, are available under https://osf.io/ndvf6/. Readers seeking access to the original, nonresampled data should contact the lead author (nina.suess@sbg.ac.at). Access will be granted in accordance with ethical procedures governing the reuse of sensitive data.

## Preregistration

The first part of the study analyses was preregistered prior to the research being conducted under https://osf.io/ndvf6/.
