# Supplementary material for: Cortical tracking of formant modulations derived from silently presented lip movements and its decline with age
Source: Cereb Cortex. 2022 Jan 22;32(21):4818–33. doi: 10.1093/cercor/bhab518 (PMC9627034; doi:10.1093/cercor/bhab518)
Supplement: Supplementary_material_cercor_bhab518 [file supplementary_material_cercor_bhab518.zip › Supplementary_material_cercor_bhab518.docx]

# Supplementary material

*Table S1: Fixed effects ANOVA results with coherence as the dependent variable*

| Type III Analysis of Variance Table with Satterthwaite's method | | | | | | |
| --- | --- | --- | --- | --- | --- | --- |
| Predictor | Sum Sq | Mean Sq | NumDF | DenDF | F-value | Pr(>F) |
| Speech features | 0.000577 | 1.9241e-04 | 3 | 346 | 32.778 | <2e-16 *** |
| Naturalness of speech | 0.000074 | 7.3518e-05 | 1 | 346 | 12.524 | 0.0005 |

Signif. codes: 0 '***' 0.001 '**' 0.01 '*' 0.05 '.' 0.1 ' ' 1

*Note: Table shows the output from R (R Core Team, 2021)*

Table S2: Results of the planned contrasts from the GLM with the factors *speech features* and *naturalness of speech*

| **Contrast** | **Estimate** | **SE** | **z** | **p_FDR_** |
| --- | --- | --- | --- | --- |
| lip - formant | 0.0011771 | 0.0003426 | 3.435 | 0.00071 *** |
| pitch - formant | -0.0021708 | 0.0003426 | -6.336 | 7.09e-10 *** |
| speech - formant | -0.0002641 | 0.0003426 | -0.771 | 0.44087 |
| pitch - lip | -0.0033479 | 0.0003426 | -9.771 | < 2e-16 *** |
| speech - lip | -0.0014412 | 0.0003426 | -4.206 | 3.90e-05 *** |
| speech - pitch | 0.0019068 | 0.0003426 | 5.565 | 5.25e-08 *** |

*Note: lip = lip-brain coherence, formant = unheard formant-brain coherence, speech = unheard speech envelope-brain coherence, pitch = unheard pitch-brain coherence*


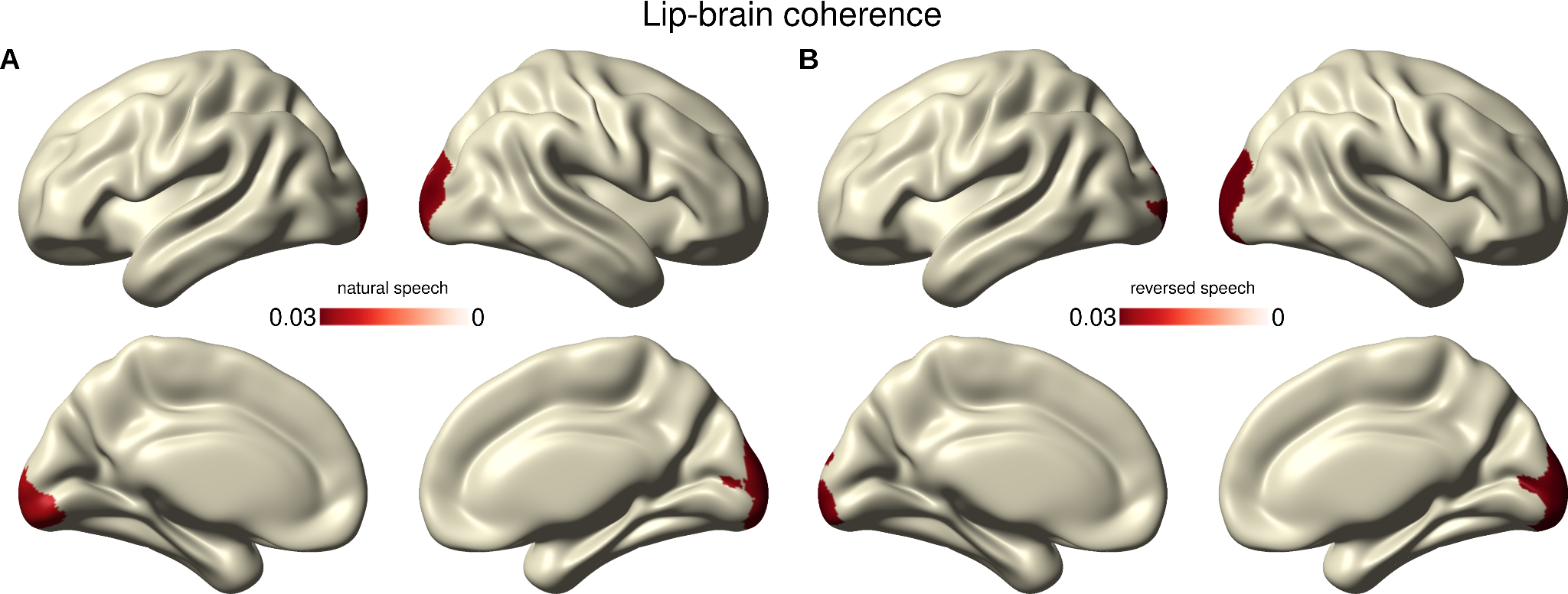


*Figure S1: Grand average of coherence values between lip movements and brain data. A) Lip-brain coherence for natural (forward) visual speech showing highest values in occipital areas (GA_max_ = 0.032, MNI [30 -104 30]). A) Lip-brain coherence for reversed (backward) visual speech showing also highest values in occipital areas (GA_max_ = 0.033, MNI [20 -90 10]). Marked voxels reached at least a threshold of 90% of the whole-brain maximum coherence value. GA_max_ = maximum coherence value*


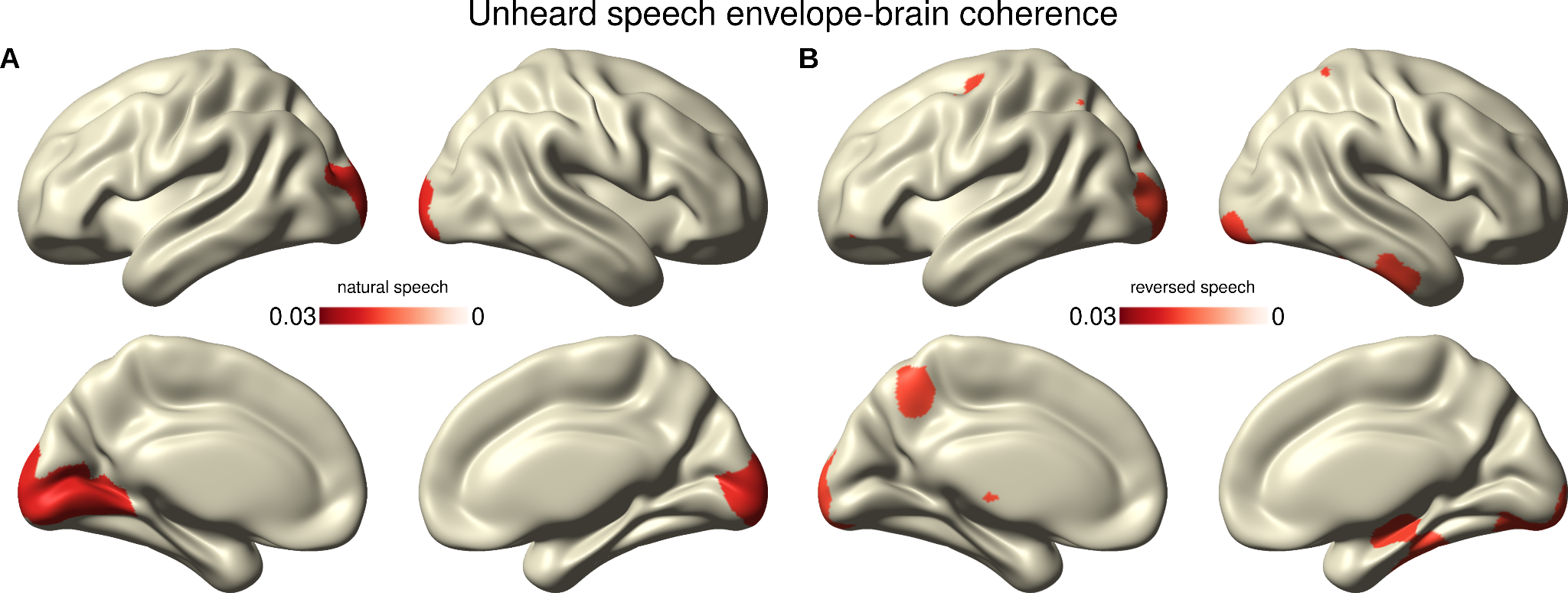


*Figure S2: Grand average of coherence values between unheard speech envelope and brain data. A) Unheard speech-brain coherence for natural (forward) visual speech showing highest values in occipital areas (GA_max_ = 0.022, MNI [-20 -110 0]). A) Unheard speech-brain coherence for reversed (backward) visual speech showing also highest values in occipital areas (GA_max_ = 0.020, MNI [30 -102 -20]). Marked voxels reached at least a threshold of 90% of the whole-brain maximum coherence value. GA_max_ = maximum coherence value*


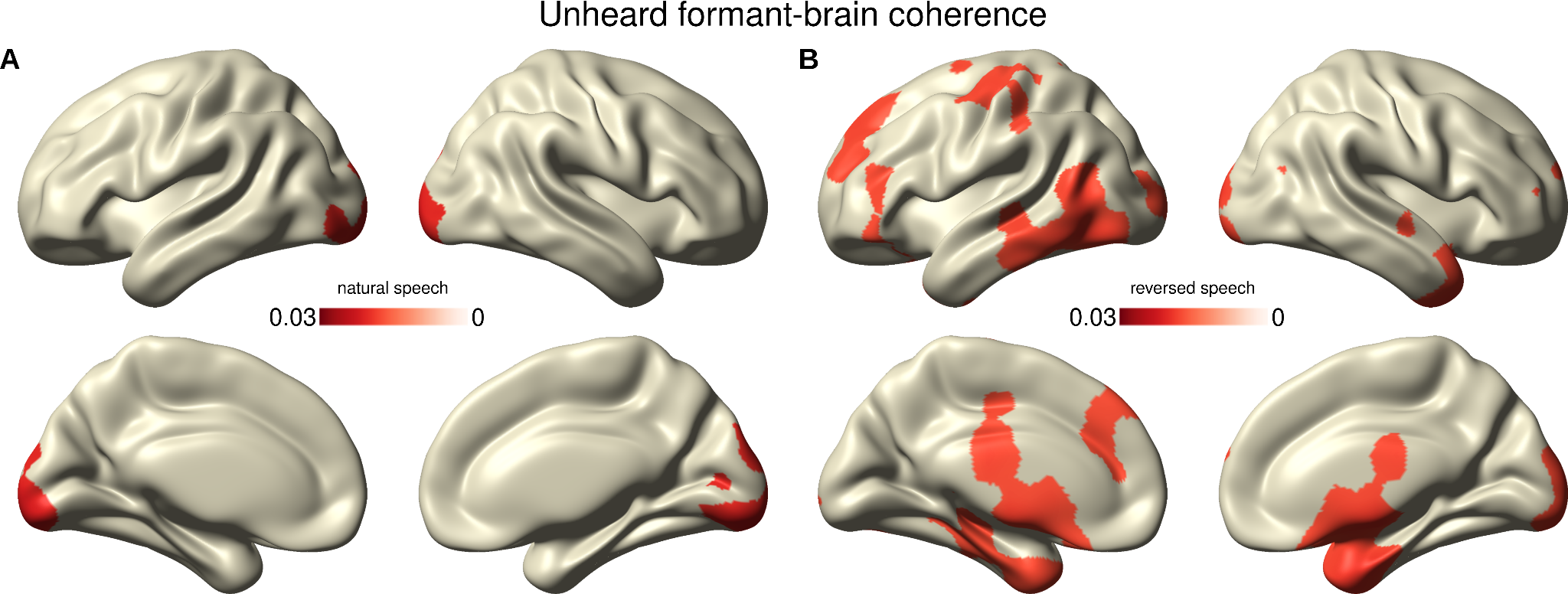


*Figure S3: Grand average of coherence values between unheard formants and brain data. A) Unheard formant-brain coherence for natural (forward) visual speech showing highest values in occipital areas (GA_max_ = 0.023, MNI [-20 -100 -10]). A) Unheard formant-brain coherence for reversed (backward) visual speech showing widespread activation, with highest values in brainstem areas (GA_max_ = 0.020, MNI [0 -20 -50], not shown here). GA_max_ for reversed speech at the MNI coordinates from A is markedly reduced (0.017). Marked voxels reached at least a threshold of 90% of the whole-brain maximum coherence value. GA_max_ = maximum coherence value*


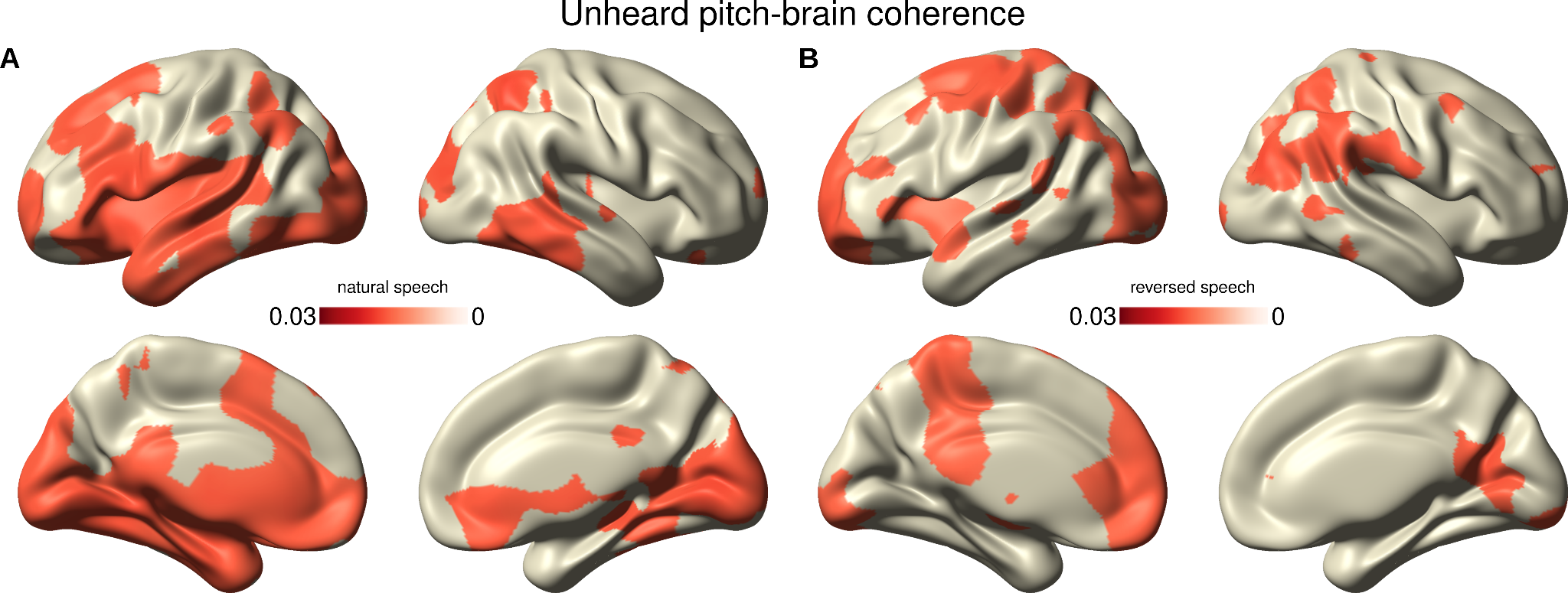


*Figure S4: Grand average of coherence values between unheard speech envelope and brain data. A) Unheard formant-brain coherence for natural (forward) visual speech showing widespread activation with highest values in posterior parietal areas (GA_max_ = 0.018, MNI [30 -60 60]). A) Unheard formant-brain coherence for reversed (backward) visual speech showing widespread activation, but highest values in occipital voxels (GA_max_ = 0.018, MNI [40 -80 20]). Marked voxels reached at least a threshold of 90% of the whole-brain maximum coherence value. GA_max_ = maximum coherence value*

*
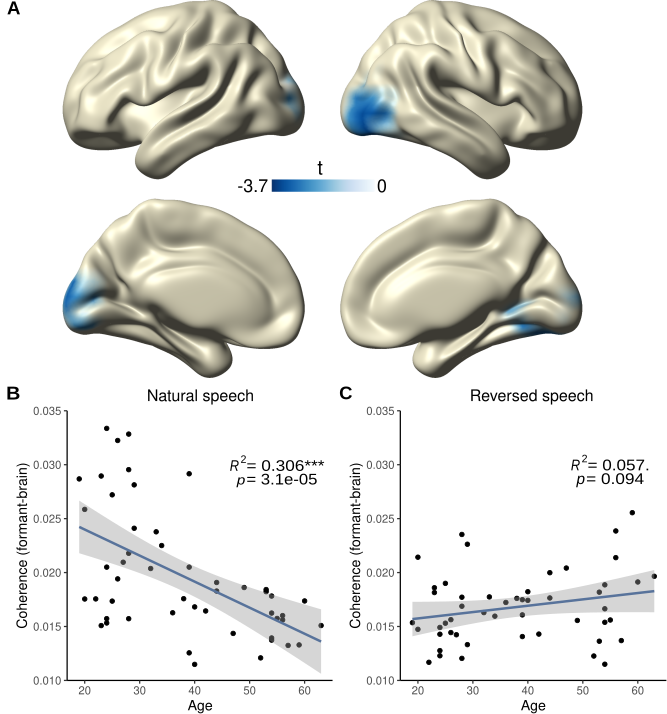
*

*Figure S5: A) Statistical values of the voxelwise correlation of the natural (forward) unheard formant-brain coherence with age (averaged over 1-7 Hz, p < 0.05, cluster-corrected). B) Correlation of unheard formant-brain coherence in significant occipital voxels extracted from A showing a significant correlation with age (p = 3.1e-05). C) Correlation of unheard reversed (backward) formant-brain coherence with age in significant occipital voxels extracted from A showing a reverse trend to the natural (forward) condition in B (p = 0.094).*
